# Modified Isoinertial-Based Ruffier Test in Healthy Individuals: A Feasibility Study

**DOI:** 10.3390/jfmk8020036

**Published:** 2023-03-24

**Authors:** Bruno Trovato, Federico Roggio, Luca Petrigna, Giuseppe Musumeci

**Affiliations:** 1 Department of Biomedical and Biotechnological Sciences, Section of Anatomy, Histology and Movement Science, School of Medicine, University of Catania, Via S. Sofia n°97, 95123 Catania, Italy; 2 Sport and Exercise Sciences Research Unit, Department of Psychology, Educational Science and Human Movement, University of Palermo, Via Giovanni Pascoli 6, 90144 Palermo, Italy; 3 Research Center on Motor Activities (CRAM), University of Catania, Via S. Sofia n°97, 95123 Catania, Italy; 4 Department of Biology, Sbarro Institute for Cancer Research and Molecular Medicine, College of Science and Technology, Temple University, Philadelphia, PA 19122, USA

**Keywords:** cardiorespiratory fitness, exercise testing, validity, isoinertial, sport science

## Abstract

Cardiorespiratory fitness is an essential indicator in sports science and sports medicine that can be assessed with several tests. The Ruffier test is a submaximal test valid to estimate maximum oxygen uptake; it consists of 30 squats in 45 s, which may be challenging for inexperienced individuals. This study aims to verify the feasibility of a modified inertial-based version of the Ruffier test to assess cardiorespiratory fitness with 10 squats in 15 s. Both classic and isoinertial Ruffier tests were administered to thirty-five healthy young adults (20 men and 15 women), age 22.06 ± 2.13 years, BMI 23.87 ± 2.74. The two one-sided test confirmed the comparability of the isoinertial Ruffier test with its classic version within equivalence bounds of ±3.726. Furthermore, gender, age, body weight, the difference between peak heart rate after isoinertial squatting and resting heart rate, and the isoinertial Ruffier index are the coefficients of our best VO_2_max prediction model with an adjusted R^2^ of 0.937, sensitivity of 0.89, and specificity of 0.81. The study evidenced the feasibility of the isoinertial Ruffier test to measure cardiorespiratory fitness through a quick, safe, and short squat test easy to perform in fitness centers and primary care clinics.

## 1. Introduction

Physical fitness is considered a marker of health status [1]; it can be assessed through valid, reliable, and feasible laboratory or field tests [2,3,4,5]. Cardiorespiratory endurance, one of the components of physical fitness [6], can be evaluated with field tests, for example, the Cooper test [7] or the 20 m Shuttle Run Test [8] or through laboratory evaluation on a treadmill wearing masks [9]. Its valuation is an important element in the prevention; indeed, cardiorespiratory fitness (CRF) strongly predicts cardiovascular events and mortality [10]. Low levels of CRF can lead to an increase in all-cause mortality in men and women [11]. An unhealthy lifestyle with the consumption of tobacco, an unhealthy diet, and low levels of physical activity can put a person at risk of developing heart disease and metabolic syndrome, and these problems are common in young people due to young age [12]. Thus, it is important to expand the evaluation of the CRF parameter to as many people as possible, regardless of age.

Despite the great importance of cardiorespiratory fitness as a recognized marker of cardiovascular disease, this parameter is often underestimated in routine clinical examinations [13]. The best index of CRF is the maximum oxygen uptake (VO_2_max), which gives information about the maximal aerobic power of an individual [14]. The evaluation of VO_2_max requires appropriate equipment, and the tests needed to measure it are not sustainable for nonathletes because they do not meet the sub-maximal exercise criteria [15]. Many submaximal protocols have been designed through the years employing different activities such as walking, cycling, running, and stepping, all feasible in laboratories or fitness centers [16] but not in routine clinic visits. Standard protocols to predict VO_2_max require at least 4/6 min of physical exercise; this demanding use of time can discourage clinicians from evaluating CRF in primary care. There are different VO_2_max evaluation methods that requires a minimum amount of time and no special equipment such as the Chester step test [17], and the incremental shuttle walking test [18]. Between these methods to evaluate cardiorespiratory fitness with no equipment, there is also a squat test named the Ruffier test. Different studies assessed its validity in the prediction of VO_2_max [19,20,21,22]. The subject has to perform 30 squats in 45 s, following a metronome set at 80 bpm and measuring the heart rate (HR) at the end of the test and one minute after the end. However, this test may be challenging for those who are unable to correctly perform the squat movement following an imposed rhythm and without assistance. Squat movement is often considered an accessible exercise for everyone, especially since it is prescribed in rehabilitation programs [17]. However, there is variability in squat performance due to gender, level of experience, and specific anthropometrics of the individuals with differences in kinematics and timing [23]. This could expose people to wrong execution and thus not meet the expected results.

Isoinertial machines in health care have increased over the years due to their versatility. The isoinertial system is independent of gravity, and it is based on a flywheel technology providing also an eccentric overload [24]. It uses the moment of inertia of a wheel in rotation during the concentric phase while slowing to resist the kinetic energy collected until stopping the wheel at the end of the eccentric phase [25]. Resistance is proportional to acceleration, allowing the exercise to be conducted in a continuous state of acceleration/deceleration [26]. The characteristics of safety, simplicity of operation, and almost absent risk of trauma support the use of isoinertial machines in health care and rehabilitation [27]; moreover, with these machines it is possible to train different districts of the body, giving also a reduction of the cost for those who implement it in their practice. The torque moment of the system is capable of guiding the subject with a cyclic rhythm, supporting the correct execution of a movement, and stimulating balance [28]. This training method does not work with the percentage of one-repetition maximum allowing a precise monitoring of the performance and neuromuscular fatigue, maximizing the training [29,30]. Their employment ranges from pure training and injury prevention to neurological rehabilitation [27,31,32].

Flywheel technology could be used side by side with the traditional training; hence, it is interesting to consider this machine also for testing. Furthermore, it is to consider that fitness centers often do not have gas analyzers due to their costs and time needed to perform the exam; consequently, an indirect method to extrapolate the VO_2_max have to be considered to enlarge the assessment of CRF to enhance the general health prevention.

The objective of the present study was to study the feasibility of a quick and efficient version of the Ruffier test to assess cardiorespiratory fitness using isoinertial machines, reducing exercise volume and execution time, in a sample of healthy young adults. To the best of our knowledge, at the moment the literature does not provide a squat test for the assessment of CRF that minimize the bias related to the differences in each subject in performing the squat movement in terms of rhythm and kinematics. Executing the Ruffier test correctly may be challenging for people with no experience in squatting movement; however, with an isoinertial machine due to the mechanics of the isoinertial exercise the subjects can keep the correct shape of the body, avoiding incorrect movements and maintaining the correct pace throughout the test. Hence, the modified inertial-based version proposed in this study could represent an alternative to the original Ruffier test to assess VO_2_max quickly and safely in routine clinical examinations. Moreover, considering the spread of isoinertial machines also in fitness centers, this test could be a feasible, harmless, and quick way to assess CRF for prevention in the young population.

## 2. Materials and Methods

Fifty young adults (30 men and 20 women) were recruited voluntarily at the Research Center on Motor Activities (CRAM), University of Catania. The inclusion criteria to meet for the participants were an age between 18 and 25, no cardiovascular disease, the ability to squat to a knee angle of 90°, and the ability to follow a rhythm of 80 bpm while squatting. Thirty-five of them (20 men and 15 women) were eligible for the study, and 15 were excluded because they met the preselected exclusion criteria consisting of recent joint trauma of the lower limb (n = 2), pain during squatting (n = 1), neurodegenerative disease (n = 1), musculoskeletal diseases (n = 1), musculoskeletal injuries (n = 2), presence of internal medical devices (e.g., pacemaker, hearing aid and similar) (n = 3), inability to squat properly (n = 3), inability to follow the 80 BPM rhythm (n = 2), pregnancy (n = 0), and heart diseases (n = 0). The average age of our sample was 22.06 years (Standard deviation (SD) = 2.13), mean body mass index (BMI) 23.87 (SD = 2.74), and mean VO_2_max 42.39 (SD = 8.08) evaluated with the classic version of the Ruffier test. The study was approved by the Research Center on Motor Activities (CRAM) Scientific Committee (Protocol n.: CRAM-022021, 20 December 2021), in accordance with the declaration of Helsinki. Informed consent was obtained for the publication of identifying information/images in an online open-access publication. Before testing, all participants provided written informed consent. A researcher collected baseline information from each participant, including age, gender, height, and body weight, then described the study protocol. Participants were instructed to perform the Ruffier test, that is, 30 bodyweight squats in 45 s and then resting for 30 min after completion. Afterward, they performed the isoinertial Ruffier test, 10 squats in 15 s, using the isoinertial machine.

### 2.1. Isoinertial Machine

**Figure 1:**
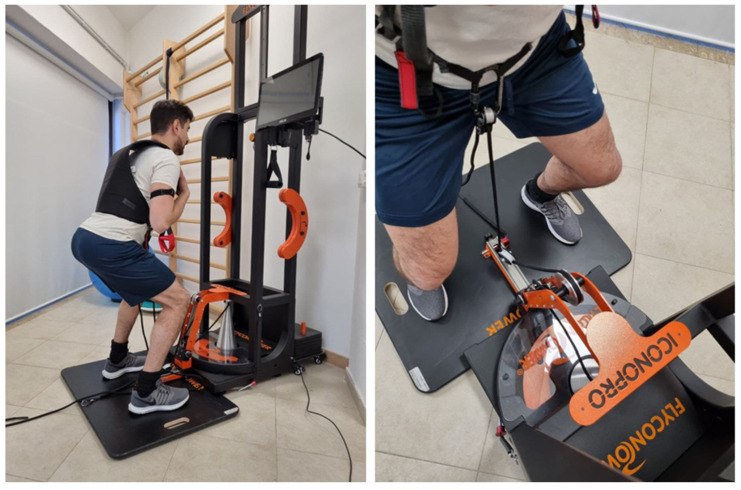
A subject performing squats with an isoinertial machine.

Isoinertial machines apply the concept of a yo-yo system by using the principle of kinetic energy accumulation on a flywheel [24]. A rope is wrapped around the cone, which stores kinetic energy during the athlete’s concentric movement phase. The length of the rope is measured to rewind the cone when the movement is completed. The individual has to oppose an eccentric braking force that slows the rotational inertia of the flywheel and allows the subsequent cyclic movement. In isoinertial training, resistance is generated by a mass placed in rotation; the athlete works against his inertia force. The Flyconpower conical machine (Cuneo, Italy) was used to administer the isoinertial Ruffier test. The participants were attached to the flywheel device by a rope fixed to their waist, while the pulley was perpendicular to the ground to freely perform the squat movement, Figure 1.

### 2.2. Experimental Setup

Before the tests, the researcher attached a Polar^®^ OH1 armband to the upper right arm of the participant and instructed them to rest for 15 min. The resting HR was collected at the end of 15 min (P1) as suggested by the literature [33]. Subsequently, the participant had to perform 30 air squats in 45 s. HR was collected immediately after the test (P2). The participant rested for one minute; then, the HR was collected again (P3). After 30 min, the participant performed the isoinertial Ruffier test, namely 10 squats in 15 s, using the isoinertial machine. HR was collected immediately after the isoinertial test (P2-i). The participant rested for one minute; then, the HR was collected again (P3-i). Based on the HR features, we calculated the Ruffier Index (RI) and the Ruffier-Dickson Index (RDI): RI = (P1 + P2 + P3 − 200)/10 and RDI = ((P2 − 70) + 2 · (P3 − P1))/10. We also calculated the isoinertial Ruffier Index (RI-i) and the isoinertial Ruffier-Dickson Index (RDI-i) with the data obtained from the isoinertial Ruffier test by replacing P2 and P3 with P2-i and P3-i. The classic Ruffier test and its isoinertial version were performed randomly.

### 2.3. Equivalence Test

We calculated VO_2_max for both the classic Ruffier test and the isoinertial Ruffier test using the Guo et al. model [34]. The primary outcome was to observe the comparability of both tests, so we assessed their equivalence using the two-one-sided test (TOST) method [35]. Specifically, the equivalence is met when the lower and upper boundaries of the 90% confidence interval (CI) are less than the defined equivalence margins. The lower (ΔL) and upper (ΔU) equivalence boundaries were set as −0.46 and + 0.46 Cohen’s d. The alternative hypothesis is ΔL ≤ μ1 − μ2 ≤ ΔU, where μ1 − μ2 represents the difference in mean scores between the two VO_2_max measurements, while the null hypothesis is μ1 − μ2 < ΔL or μ1 − μ2 > ΔU. If the null hypothesis is rejected, the equivalence is present; the two tests produce the same outcome. We used the Two-sample TOST power calculation to evaluate the power analysis of the results.

### 2.4. Multiple Linear Regression Models

We used the anthropometric characteristics of the participants, HR measurements, RRI, and RDI to build three different multiple linear regression models to predict the VO_2_max of the isoinertial Ruffier test. In model 1, we used participants’ characteristics and H.R. measurements; in model 2, we used participants’ characteristics, H.R. measurements, and RI-i; in model 3, we used participants’ characteristics, H.R. measurements, and RDI-i. To assess the quality of our models, we calculated sensitivity, specificity, and Cohen’s kappa. Following Sartor et al. [36], we classified CRF levels into three categories based on the American College of Sports Medicine (ACSM) norms. We pooled ACSM classification into poor (ACSM’s very poor and poor), fair (ACSM’s fair), and good (ACSM’s good, excellent, and superior) with different parameters for males and females. Sensitivity was computed as follows: TPc/(TPc + FNc), where TPc represents the number of true-positive cases where the model adequately predicted fitness levels fair and good; FNc represents the number of false-negative cases where the model underestimated the fitness levels. Specificity was computed as TNc/(TNc + FPc), where TNc represents the number of true negative cases where the model adequately predicted fitness level poor; FPc represents the number of positive cases where the model overestimated the fitness levels. Furthermore, we computed Cohen’s kappa [37] to measure the agreement between the VO_2_max model of Guo et al. [34] and our models.

We used the leave-one-out cross-validation (LOOCV) method to evaluate the stability of our models. In the LOOCV, one participant is removed from the sample, and then the software builds a VO_2_max prediction model. Subsequently, it uses the data from the removed participant to validate the prediction model; it is repeated for all participants. The results of this method are the root mean squared error (RMSE), which represents the average difference between the actual observations and the predictions made by the model, and the mean absolute error (MAE), which represents the average absolute difference between the actual observations and the predictions made by the model.

## 3. Results

We noted all the characteristics of the participants in Table 1. The male participants had a mean VO_2_max of 46.6 (6.0) ml·kg^−1^·min^−1^, while the mean of the female participants was 36.78 (7.13) ml·kg^−1^·min^−1^. The mean VO_2_max referred to as isoinertial was 46.16 (5.70) and 36.29 (7.50) ml·kg^−1^·min^−1^ for men and women. H.R. measurements of the classic Ruffier test and the isoinertial one are reported in Table 1.

**Figure 2:**
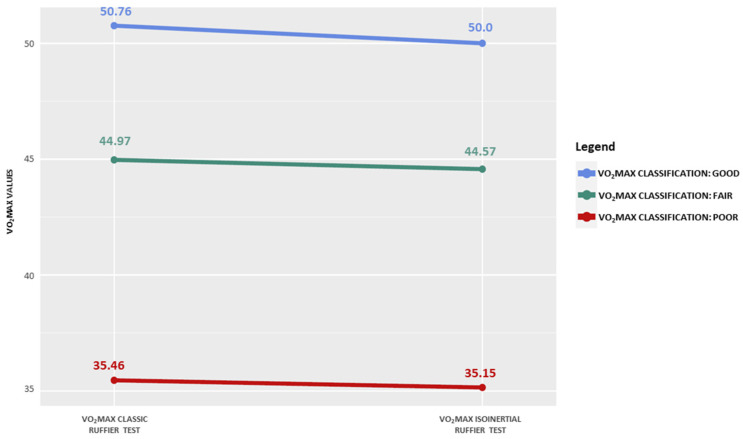
Slope chart comparing the VO_2_max of classic and isoinertial Ruffier tests.

**Figure 3:**
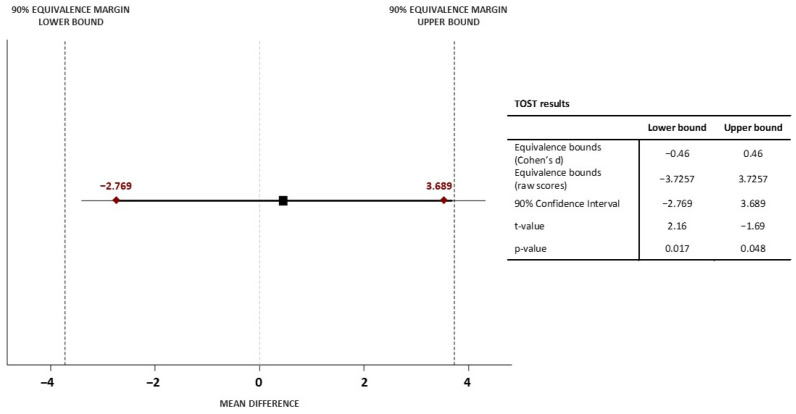
Ninety percent confidence interval of the mean difference in VO_2_max between classic and isoinertial Ruffier test.

The first objective was to observe the comparability of the VO_2_max values deriving from the two different Ruffier test execution methods. Analysis of the primary outcome carried out with the TOST method indicates that the classic Ruffier test VO_2_max, namely 30 squats in 45 s, is equivalent to the isoinertial Ruffier test VO_2_max, 10 squats in 15 s with the isoinertial system. The mean VO_2_max of the classic Ruffier test was 42.39 (8.08) ml·kg^−1^·min^−1^ while the mean of the Ruffier isoinertial test was 41.93 (8.11) ml·kg^−1^·min^−1^. As reported in Figure 2, the mean difference in VO_2_max divided into poor, fair, and good between the two tests is small. The lower and upper boundaries set on Cohen’s d were ±3.726. The test result reported lower and upper boundaries with a 90% CI of −2.769 and 3.689, respectively. The equivalence boundaries fall within the selected limits, so we can conclude that the observed effect is statistically not different from zero, namely statistically equivalent to zero, Figure 3. According to the mean difference, SD of difference, and lower and upper bounds, the power analysis provided a value of 0.99.

The second objective was to analyze the results obtained using a multiple linear regression method. We noted all the results of the three models for predicting VO_2_max in Table 2. All three methods were significant in predicting VO_2_max (*p* < 0.001). The coefficients of model 1 are gender, age, body weight, P2-i − P1, and P2-i − P3-i. This model has an adjusted R^2^ of 0.876 with 0.94 sensitivity and 0.76 specificity. Model 3′s coefficients are gender, age, body weight, P2-i − P1, and RI-i. This model has an adjusted R^2^ of 0.937 with 0.89 sensitivity and 0.81 specificity. Model 3′s coefficients are gender, age, body weight, P2-i − P1, and RDI-i. This model has an adjusted R^2^ of 0.910 with 0.94 sensitivity and 0.82 specificity. Both models 2 and 3 have high validity to predict VO_2_max. To assess the best model to perform the prediction, we also performed the LOOCV. Model 2 presents a lower NRMSE 0.052 and a lower NMAE 0.043 than the NRMSE 0.063 and NMAE 0.049 of model 3. Generally, all three models have small prediction errors relative to the mean VO_2_max (42.39 mL·kg^−1^·min^−1^), but we point out model 2 as the best to predict VO_2_max.

Therefore, when administering the isoinertial Ruffier test, 10 squats in 15 s, the model to predict VO_2_max according to our best model is:

VO_2_max = 103.096 − 15.927 × (gender) − 0.785 × (age) − 0.560 × (body weight) + 0.172 × (P2-i − P1) − 0.806 × (RI-i)

where gender is coded as 0 for men and 1 for women; body weight is expressed in kilograms (kg); P2-I P1 is the difference between the maximum HR after squatting; and the resting HR, RI-i is the formula (P1 + P2-i + P3-i − 200)/10; these last two values are expressed as beats·min^−1^.

## 4. Discussion

This study proposed a modified inertial-based Ruffier test to assess cardiorespiratory fitness by reducing the time needed to perform the test and employing an isoinertial machine. To our knowledge, this is the first study to employ isoinertial exercise in a cardiorespiratory fitness test to measure VO_2_max. The increasing presence of isoinertial machines in clinics and fitness centers provides advantages in terms of equipment costs and safety for the patient due to its intrinsic mechanics of functioning [27] making this test ideal to assess the CRF. The analysis of the equivalence of VO_2_max analysis between the classic Ruffier test and the isoinertial Ruffier test and the building of three multiple regression models based on anthropometric values, HR measurements, Ruffier index, and Ruffier-Dickson index formulas to predict VO_2_max using isoinertial measurements could be useful for the community to understand the rationale of this modified test version.

The oxygen consumption evaluated using the Ruffier-Dickson test has a moderate to vigorous impact (~6 peak MET) on the aerobic system and cardiorespiratory load [36]. The Guo et al. VO_2_max prediction formula [34] was used to evaluate that value from the classic Ruffier test and modified isoinertial-based Ruffier test. The classic version of this test may be challenging because it requires a discrete ability to correctly perform the movement. Furthermore, the subject must strictly follow a fast pace to perform 30 repetitions in 45 s, which may negatively affect test reproduction. The basic squat movement is highly sensitive to emphasize biomechanical deficits such as muscle weakness, motor unit recruitment, joint asymmetry, and instability [38,39]. Based on this assumption, we reduced the execution time from 45 to 15 s, and we employed the flywheel system in order to support the cadence of the movement and guide the participant toward a flowing movement performance. We provided for the first time a modified version that includes the use of an isoinertial machine that helps the subject keep a constant pace, reducing the number of repetitions from 30 to 10 and the total time from 45 to 15 s. Within a 90% CI, our results showed that both VO_2_max predictions are equivalent according to the TOST method. Therefore, the isoinertial Ruffier test, 10 squats in 15 s, is a feasible, short, and quick alternative to the classic Ruffier test.

Once we assessed the feasibility of the isoinertial Ruffier test, three multiple linear regression models to predict VO_2_max through isoinertial HR measurements were built. The adjusted R^2^ among the models was lower (model 1 = 0.876, model 2 = 0.937, model 3 = 0.910), but the best performing model is model 2, which comprises the Ruffier formula, adapted to the isoinertial method (RI-i). Our analysis showed that the best VO_2_max prediction model was based on gender, age, body weight, P2-i − P1 (difference between the peak HR after squatting and resting HR), and the RI-i formula (P1 + P2-i + P3-i − 200)/10). This model predicted individuals’ CRF with an adjusted R^2^ of 0.937, a sensitivity of 0.89, and a specificity of 0.81. Furthermore, the data obtained from the leave-one-out cross-validation method support this model’s availability in other datasets. The errors shown are NRMSE = 0.052 and NMAE = 0.043, less than the results of Guo [34] and Sartor [36], which assessed different regression models to predict VO_2_max. Age was one of the key factors for predicting VO_2_max, in accordance with the finding of Kim et al. [40], where age was negatively correlated with VO_2_max. We also observed that VO_2_max was higher in men compared to women, as already shown in the study by Koons et al. [41]. The gender variable is important to consider as aerobic capacity measured by VO_2_max has been found to be different between males and females by previous studies [42,43]. Resting HR is undoubtedly a key value for predicting VO_2_max but, more generally, a critical value as a risk factor for mortality independently of physical fitness, leisure time, and major cardiovascular risk factors [44]. We used resting HR (P1) combined with the peak HR after squatting (P2-i) because it showed the best significance for the regression models. Our results differ from those of Guo et al. [34], who found height to be a valuable predictor of normalized VO_2_max by resting HR, but also from Mohammed et al. [45], who found height to be a strong predictor of maximal aerobic capacity. Finally, body weight is a strong coefficient of our model. It is in line with several studies that observed how body weight could influence cardiorespiratory fitness [46,47]. Specifically, Hung et al. stated that an increase in the BMI value of one unit could lower cardiorespiratory fitness by 0.316 and 0.368 points for adult males and females, respectively. Compared to the famous and reliable 6 min walking test [48], our best model of prediction of the VO_2_max has similar predictors such as age, body weight, and heart rate post-exercise, confirming that the parameters that entered our model are also considered suitable in this CRF test. Moreover, our best model of VO_2_max prediction had an r^2^ of 0.937, similar to the r^2^ detected in the study of Mänttäri et al. [48] for the 6 min walking test (r^2^ = 0.85 in males, 0.90 in females) and in the study of Björkman et al. [49] for the Ekblom Bak test (r^2^ = 0.86 in males, 0.83 in females). However, we consider to interpretate these findings with cautions considering that, conversely to the studies previously cited, we did not employ a gas analyzer.

This study has strengths and limitations. The main strength is that our version of the Ruffier test requires less time and effort than its classical version, and it does not require gas analyzer; it could also be potentially used in a broader range of people, considering the risk of injuries when performing isoinertial exercise almost non-existing [27]. In contrast, we know that all participants in our samples were young and healthy. Furthermore, people with heart disease or musculoskeletal disorders may not perform the test correctly or with comparable results. Furthermore, we did not compare our data with the gold standard measurement for VO_2_max assessment with a metabolic analyzer during a submaximal test. Future studies on healthy subjects from different age groups (e.g., adolescents, elderly) may be carried out to evaluate the feasibility for this test also in this populations. Moreover, future studies should focus on the possibility of employment of the isoinertial Ruffier test in patients with various clinical conditions to find a more suitable way to assess CRF in people with clinical conditions that may benefit from it.

## 5. Conclusions

For the first time in the literature, we proposed a modified inertial-based Ruffier test executed with an isoinertial machine, a short and fast CRF carried out in 15 s with 10 squat repetitions. Furthermore, we advise an isoinertial VO_2_max prediction model based on anthropometric parameters, HR measures, and the Ruffier index formula. Our best model includes HR characteristics (P2-i − P1) and the isoinertial Ruffier index (RI-i = P1 + P2-i + P3-i − 200)/10). This study provides information about the reproducibility and validity of isoinertial machines to assess cardiorespiratory fitness. The proposed modified inertial-based version of the Ruffier test can predict VO_2_max reasonably well in healthy males and females. Future studies concerning the validity of isoinertial machines will be carried out by analyzing different movements in the fields of clinic and sports medicine.

## Figures and Tables

**Table 1:** Participants’ characteristics.

	Overall n = 35 Mean (SD)		Men n = 20 Mean (SD)		Women n = 15 Mean (SD)	
Age (years)	22.06	(2.13)	21.7	(1.69)	22.53	(2.59)
Height (cm)	170.46	(9.56)	176.45	(5.17)	162.47	(8.13)
Weight (kg)	69.57	(11.19)	75.35	(8.63)	61.87	(9.58)
BMI (Kg/m^2^)	23.87	(2.74)	24.21	(2.56)	23.41	(2.99)
P1 (beats·min^−1^)	69.57	(9.93)	67.15	(10.4)	72.8	(8.56)
P2 (beats·min^−1^)	138.09	(15.58)	133.45	(12.84)	144.27	(17.16)
P3 (beats·min^−1^)	106.14	(17.42)	105.25	(14.82)	107.33	(20.88)
P2-i (beats·min^−1^)	135.23	(14.57)	131.55	(13.06)	140.13	(15.45)
P3-i (beats·min^−1^)	105.97	(18.74)	105.25	(18.67)	106.93	(19.44)
VO_2_max (ml·kg^−1^·min^−1^)	42.39	(8.08)	46.6	(6)	36.78	(7.13)
VO_2_max-i (ml·kg^−1^·min^−1^)	41.93	(8.11)	46.16	(5.7)	36.29	(7.5)
RI-i	11.08	(3.62)	10.39	(3.47)	11.99	(3.75)
RDI-i	69.01	(22.98)	68.88	(22.94)	69.2	(23.83)

BMI = body mass index; P1 = resting heart rate before squatting; P2 = peak heart rate after squatting; P3 = recovery heart rate 60 s after squatting; RI = Ruffier index; RDI = Ruffier-Dickson index; P2-i = peak heart rate after isoinertial squatting; P3-i = recovery heart rate 60 s after isoinertial squatting; RI-i = isoinertial Ruffier index; RDI-i = isoinertial Ruffier-Dickson index.

**Table 2:** Multiple linear regression models to predict VO_2_max.

	Multiple Linear Regression							LOOCV			Model Performance		
	B	SE	t	β	*p*	R^2^	Adj. R^2^	NRMSE	r^2^	NMAE	Sensitivity	Specificity	*k*
**Model 1**						0.89	0.87	0.07	0.84	0.06	0.94	0.76	0.77
Intercept	93.36	6.73	13.86		<0.001								
Gender	−17.28	1.29	−13.3	−1.07	<0.001								
Age	−0.78	0.24	−3.22	−0.2	<0.01								
Weight	−0.51	0.05	−9	−0.7	<0.001								
P2-i − P1	0.07	0.03	2.09	0.22	<0.05								
P2-i − P3-i	0.15	0.04	3.51	0.12	<0.01								
**Model 2**						0.94	0.93	0.05	0.92	0.04	0.89	0.81	0.81
Intercept	103.09	4.8	21.46		<0.001								
Gender	−15.92	0.92	−17.3	−0.98	<0.001								
Age	−0.78	0.17	−4.52	−0.2	<0.001								
Weight	−0.56	0.04	−13.75	−0.77	<0.001								
P2-i − P1	0.17	0.02	6.42	0.31	<0.001								
RI-iso	−0.80	0.11	−7.17	−0.36	<0.001								
**Model 3**						0.92	0.91	0.06	0.88	0.04	0.94	0.82	0.82
Intercept	95.33	5.67	16.80		<0.001								
Gender	−17.01	1.09	−15.50	−1.05	<0.001								
Age	−0.78	0.20	−3.77	−0.20	<0.001								
Weight	−0.52	0.04	−10.84	−0.72	<0.001								
P2-i − P1	0.29	0.04	6.01	0.54	<0.001								
RDI-iso	−0.16	0.03	−5.26	−0.46	<0.001								

BMI = body mass index; P1 = resting heart rate before squatting; P2-i = peak heart rate after squatting; P3-i = recovery heart rate 60 s after isoinertial squatting; RI-i = isoinertial Ruffier index; RDI-i = isoinertial Ruffier-Dickson index; SE = standard error; LOOCV = leave-one-out cross-validation; NRMSE = normalized root-mean-square error to mean of the VO_2_max; NMAE = normalized mean absolute error to mean of the VO_2_max; *k* = Cohen’s kappa.

## Data Availability

All data are available from the corresponding author on reasonable request.
